# Entrepreneurial activities in policy implementation: Sweden’s national wind coordinators

**DOI:** 10.1007/s10113-018-1286-x

**Published:** 2018-02-14

**Authors:** Sarah Giest

**Affiliations:** Institute of Public Administration, Faculty of Governance and Global Affairs, Leiden University, Turfmarkt 99, 2511 DP The Hague, The Netherlands

**Keywords:** Climate governance, Wind power, Entrepreneur, Entrepreneurship, Policy implementation

## Abstract

A growing body of literature focuses on how the context in which policy entrepreneurs operate shapes their actions. This study contributes to this perspective by focusing on the regional implementation of wind turbines for increasing renewable energy levels in Sweden. Sweden introduced national wind coordinators for facilitating wind energy implementation. In this capacity, the coordinators carry out entrepreneurial strategies in form of moving the policy through the administrative agenda at local level and pursuing the implementation process together with municipal stakeholders. The study shows that over time, wind coordinators were able to move beyond the government-defined activities and widen the scope of their actions. The analysis offers insights into the temporal dimension of regional entrepreneurial activities by mapping activities from 2006 to 2016. The case reveals that a flexible policy framework and more in-depth knowledge into regional struggles through mediating and networking enable the identification of potential local bottlenecks and lobbying for legal changes by entrepreneurs.

## Introduction

The Swedish government is using wind coordinators to enhance local wind power levels in line with the goal of increasing its share of renewable energies in gross domestic energy consumption from 32% in 2008 to 50% in 2020. The Ministry of Environment and Energy established a wind power network and “national wind coordinators” (vindkraftordnarnas) with responsibilities for certain regions (Söderholm and Pettersson 2011). These wind coordinators take on some of the public engagement activities and investment negotiations. The four coordinators were appointed in 2006 and together take up one full-time position, each one devoting 25% of their time to wind power implementation in the southeast, southwest, northeast, and northwest of the country. The main tasks of the coordinators, defined by government, include knowledge dissemination in connection to wind power to the public and local communities as well as distributing information about the licensing and environmental approval processes connected to setting up a wind turbine. They are also tasked with linking stakeholders in the energy field, facilitating the resolution of structural issues and external barriers of implementation, and finally to relay information back to the Ministry. The implementation phase is especially challenging for wind power distribution, because often times opposition forms around wind turbines and farms in local communities (Spowers 2000; Deegan 2002; Söderholm, Ek, and Pettersson 2007; Jobert, Laborgne, and Mimler 2007). To produce large amounts of energy, wind turbines occupy prominent places, such as coastal lines, leading to possible noise, and optical pollution.

In the Swedish context, there are additional implementation hurdles that are connected to the veto power of municipalities. In June 2006, Parliament approved a wind bill that allows municipalities, county councils, and other authorities to actively contribute to improved conditions for the planning of a community-based, renewable, and sustainable electricity production based on wind power (SME 2008). Municipalities thus play a key role in supporting national goals for wind power and installing wind power on the ground. Despite the centralized system, municipalities can however make decisions about land use (Granberg and Elander 2007; Ek et al. 2013). From an implementation perspective, a key hurdle is the ability of municipalities and individuals to veto wind turbines (OECD 2012). The Planning and Building Act (PBA) (SFS 2010:900) determines that the municipality grants or denies a construction permit for wind turbines (Rudberg et al. 2013; Swedish National Board of Housing 2016). The directive^1^ was originally intended to simplify and shorten the process of wind turbine implementation throughout Sweden, but in practice adds a legal insecurity for potential investors (Andersson 2010; Nilbecker 2014; Larsson 2014).

Looking at this case from a policy entrepreneur perspective, the government in Sweden created a space and position in which entrepreneurial activity could flourish (Boasson 2015). Climate policymaking is becoming more decentralized and polycentric, and entrepreneurs increasingly play a role in the implementation of such measures (Giest and Howlett 2012). Limited attention has however been paid to the entrepreneurial activities of actors during the implementation phase, such as public employees further down in the political hierarchy and street level bureaucrats who facilitate policy implementation and pursue innovation (Lipsky 1980; Steelman 2010). This is despite the fact that Windrum and Koch (2008) show that entrepreneurial action partially drives implementation, especially in areas where government has a rather permissive approach to the action taken (Petridou et al. 2015). Entrepreneurial activity is hereby defined as “enhancing policy influence by altering distribution of authority and information, and/or altering norms and cognitive frameworks, worldviews, or institutional logics” (Boasson and Wettestad 2014, 405). In addition, there is an increasing amount of research focusing on the context and conditions that determine entrepreneurial action. These factors include the characteristics of the policy, the network environment, and the organizational positioning of the entrepreneur (Brouwer 2013; Palmer 2015).

The paper contributes to this research by asking *how wind coordinators use their position to undertake entrepreneurial activities that potentially go beyond the indicated structural position assigned by government*. The paper applies the policy entrepreneur literature to the Swedish case of wind power implementation, where wind coordinators carry out entrepreneurial activities when connecting with investors and the local communities for wind farming. Thereby the contribution lies in supporting efforts to fill the gap in the literature on entrepreneurial activities during the implementation stages of policy by looking at the context in which entrepreneurial action is carried out and what type of activities occur (Brouwer and Biermann 2011; Petridou et al. 2015). The Swedish government was expecting—even calculating for—some entrepreneurial action, by providing a broad framework of knowledge dissemination and networking to enhance wind power implementation and creating an institutional opportunity structure for such activities to flourish. Wind coordinators, in turn, were able to use their position and expertise to push for larger changes beyond the local setting. The field of wind power thereby serves as an example for several dynamics that environmental policies face when dealing with multi-level frameworks which require local implementation.

## Analytical framework and methodology

### Analytical framework

In the context of this paper, entrepreneurs are seen as catalysts for government to facilitate policy implementation (Jordan and Huitema 2014). More specifically, the entrepreneurial role becomes available under the condition of government putting wind coordinators in a position in which they are able to take up certain activities (Boasson 2015). Thereby the question remains, *are they able to use this position to undertake entrepreneurial activities that potentially go beyond the indicated structural position assigned by government?* There is some tension that has been identified in the literature between pre-defined structures and entrepreneurial action (Boasson 2015; Fligstein and McAdam 2012). The contexts within which entrepreneurs operate can have bearing on how these actors can exercise influence over the policy making process (Palmer 2015; Mukherjee and Giest 2017). This in turn is connected to the entrepreneur’s role and whether they have formal power or need to mobilize the public or other stakeholders.

Especially in the wind energy context, implementation is often stalled due to uncertainty connected to renewable energy legislation and changing incentives by government that affect public opinion and investments by businesses (Zoll 2001; O’Bryant 2002; Söderholm, Ek, and Pettersson 2007). Reducing these uncertainties is one of the challenges a policy entrepreneur can address (Mintrom and Norman 2009). In the Swedish context specifically, the municipal level has a strong position due to its authority over land use and the veto power on local wind power installations (Söderholm, Ek, and Pettersson 2007; Ek et al. 2013). This means that local decisions play an important role in determining the amount of wind power and possible investment opportunities.

The picture emerging from this sector-specific perspective on policy entrepreneurs and their official role in the set-up of the Swedish wind energy plans suggests two dimensions in which the entrepreneurs are active: First, the vertical dimension where they connect local to national levels. They are able to alert national government to location-specific issues and can further suggest place-based solutions. And second, at the local level, they have the ability to be leaders within a certain network of stakeholders that they set-up. In the Swedish wind energy context, the entrepreneurs are individuals that are operating at local level, using political experience and regional acceptance as tools to be active in their network (Böcher 2015). This combination of placement within the political system and expertise helps them to mediate among stakeholders and identify bottlenecks for potential policy changes.

### Entrepreneurship in environmental policy implementation

In the environmental policy field, the discussion around entrepreneurship has largely been dominated by looking at “leadership,” but more recent research has been moving towards entrepreneurial traits of individuals or organizations to connect environmental problems to policy processes. The focus has been on problem solving capacities and the synthesis of existing processes for finding solutions. This allows opening up to a more diverse set of actors. Westley et al. (2013) even argue that the expanded concept of entrepreneurship in environmental studies should replace leadership altogether, because it offers a wider range of institutionally and contextually embedded change agents. The leadership literature however provides insights into the networking dynamics and individuals traits of entrepreneurs. Many of these findings identify effective coordination and leadership at local level as factors for developing the capacity to promote climate change measures (Boyle and O’Riordan 2013). Agranoff and McGuire (2001) point out that leaders play a central role in developing network cohesion by developing common goals and engaging in strategic interaction, such as brokering, while Boyle and O’Riordan (2013) point out that there is a strong perception that local government needs a clear leader that can connect national planning agencies and local needs. The leadership function serves the purpose of connecting the different stakeholders in climate change initiatives while facilitating the capacity for implementation. This can be summed up by the definition that Ostrom (2005) offers, which says that entrepreneurship is a “particular form of leadership.” Underdal (1998), Andresen, and Agrawala (2002) also highlight entrepreneurial traits in what they label “instrumental leadership.” This includes diagnosing the problem, and inventing or exploring possible solutions as well as finding support to achieve a solution.

For policy implementation in particular, there is limited attention being paid to entrepreneurs (Petridou et al. 2015). In this paper, there is an emphasis on entrepreneurs facilitating the implementation of policies and being in an exchange relationship with political leaders that are looking to enhance public support (Roberts and King 1991). The implementation in this case is different from the one attributed to the innovative idea by an entrepreneur that has been approved and is being tested (Poole and Van de Ven 1989), but rather using entrepreneurial activities to implement a larger policy. In the Swedish case, the national government further created an institutional structure that generates opportunities for wind coordinators to act entrepreneurially (Sine and David 2003). The positioning at the local level and at the heart of the implementation process brings the entrepreneur closer to the recipients of the policy and requires certain strategies to build coalitions and trust.

### Entrepreneurial activities

Entrepreneurial acts include “enhancing policy influence by altering distribution of authority and information, and/or altering norms and cognitive frameworks, worldviews, or institutional logics” (Boasson and Wettestad 2014, 405). Thereby, structural conditions can limit as well as enable some of those activities as entrepreneurs gain access to information and decision makers. Taken together, entrepreneurial mechanisms are a collection of activities that are able to change policy (Boasson 2015). There are several ways of distinguishing different entrepreneurial activities. For the analysis of Swedish wind energy policy, the framework by Roberts and King (1991) offers a categorization of four types of activities (Table 1).

**Table 1:** Entrepreneurial activities by wind coordinators in the Swedish case

Entrepreneurial activities	Definition	Wind coordinators’ activities
Creative/intellectual activities	Generate ideas, define problem/select solution and disseminate ideas	- Knowledge dissemination
		- Identification of local challenges connected to implementing wind power
Strategic activities	Formulate strategies, evolve political strategy and develop heuristics for action	- Strategies for regional wind power implementation
Mobilization and execution activities	Establish demonstration projects, cultivate bureaucratic insiders, collaborate with high profile individuals and form lobby groups	- Collaboration with public officials at local and national level
		- Wind energy events, demonstration projects
		- Establishing networks and coalitions among local stakeholders and investors
Administrative and evaluative activities	Facilitate program administration and participate in program evaluation	- Lobbying for changes in the wind turbine licensing process, trying to optimize legal andenvironmental procedures
		- Regular meetings with the Ministry to evaluate regional obstacles and progress

#### Mobilization and execution activities

Based on the plans laid out by the Swedish government, the main role of wind coordinators is to connect stakeholders and establish local networks. The expectation is that wind coordinators are able to mobilize and execute innovative ideas. This includes broad networking activities and coalition-building as well as the cultivation of media support (Petridou et al. 2015). In addition, government plans point towards the reduction of uncertainty for investors and the spreading of information on wind turbine implementation. This is largely summarized in the mobilization and execution category. The definition of these activities is further complemented by the conceptualization of linking and relational management strategies laid out in connection to Dutch water management (Brouwer and Biermann 2011). These highlight the dynamic and more fluid aspects of entrepreneurial work where linking strategies have the purpose of engaging with other parties in coalitions, projects, ideas, and policy games. Relational management further helps to manage the relational factor in policy change trajectories (Brouwer and Biermann 2011).

To successfully execute the plans in connection with these strategies, entrepreneurs establish networks. Entrepreneurial features are thus paired with contextual dynamics and a network of personal and professional people. In other words, policy entrepreneurs make use of their network to facilitate change and draw upon skills and knowledge available in that network (Mintrom and Norman 2009; Font and Subirats 2010). Networks further give insight into the perspective of other participants, such as investors or local residents (Minstrom 2000; Williams 2002).

#### Creative/intellectual and administrative/evaluative activities

Swedish wind coordinators however go beyond those activities by also employing creative/intellectual and administrative/evaluative activities. The former category describes the generation of new ideas or the transfer of ideas from other policy domains into a new context. Coordinators also get involved in administrative and evaluative activities surrounding wind power. This is done in close collaboration with “bureaucratic insiders” who become allies within government to facilitate certain plans. These contributing factors and strategies are further perpetuated by the characteristics of the innovative idea that is being proposed. According to Roberts and King (1991), the relative advantage, compatibility, trialability, observability, and complexity play a role. This means that depending on how much improvement the idea is to the existing situation, how compatible it is to existing values, how easy it is to test, how visible it is to others, and its level of complexity when being realized in the policy realm. These types of activities include, for example, lobbying for changes in the wind turbine licensing process and aiming to optimize legal and environmental procedures.

Strategic activities encompass the formulation of both long- and short-term plans as well as heuristics for action. There are however limited strategic activities in the Swedish context, because the wind coordinators act in a pre-defined context and are themselves part of an existing political strategy and vision. Linking these dimensions to the goals set out by the Swedish government for wind coordination work, it becomes clear that the main expectations focus on mobilization and execution activities. This category encompasses coordination and linking activities as well as implementing projects. These include the disseminating of knowledge on wind power, relaying information on local implementation back to government and connecting stakeholders involved in implementation. Examples of the activities outlined by the Swedish government include investment negotiations and public engagement initiatives. However, as the description of the actual activities will show, wind coordinators go beyond their defined position and use their expertise, networks, and local knowledge to change policy.

## Methodology

### Case selection

The case was selected based on the wind energy paradox that the Swedish government was able to overcome. Sweden has had very high levels of public support for wind energy, but this favorable condition did not boost installed wind capacity (Lago et al. 2009). Much of the literature around renewable energy in Sweden has focused on this by highlighting two main obstacles: a slow and cumbersome planning and permit process as well as the localized opposition to wind turbines (Bergek 2010; Pettersson et al. 2010; Larsson 2014). Faced with these challenges, the Ministry of Environment and Energy responsible for national wind power has changed the permit process for wind farms and added wind power coordinators to enhance local understanding and acceptance while also creating a point of contact for investors to make sense of the wind planning scheme. The paper argues that those wind coordinators behave entrepreneurially and have—with this activity—contributed to raising wind power capacity in Sweden. The Swedish case further serves as an explorative case for how entrepreneurs shape activities in a setting that is pre-defined by government. Following the argument made by Sine and David (2003) that institutional structure plays an important role in creating opportunities for entrepreneurial activity, the Swedish context sheds further light on such a setting. The wind coordinator position outlined by government remains flexible and activities largely take place during the implementation rather than the agenda-setting or decision-making phase. Wind coordinators are given the opportunity to carry out wind energy-related initiatives and use the structural space created to perform different types of entrepreneurial activities.

The Swedish case can further be considered a best practice, due to the combination of a multi-pronged policy approach of which the wind coordinators are one element. The government put in place a market-based certificate program and a renewable energy plan to facilitate wind power capacity. The caveat of such an approach is however the difficulty of identifying which one of the measures worked in raising capacities in recent years.

### Within-case analysis

The study conducts a within-case analysis with the goal of exploring the type of entrepreneurial activities carried out in climate governance at local and regional level, particularly in the case of public resistance and other hurdles for the implementation of wind power capacity. Thereby, the paper uses documents published by the Swedish government, the Swedish Energy Agency and municipal documents as well as documentation of the establishment and the activities of the wind coordinators in the time between 2006 and 2016. Especially Swedish policy documents and reports on wind coordinator activities (2006–2016) and secondary literature engaging with the Swedish wind energy context have been examined. These include policy documents by the Swedish national and local government, the Swedish Energy Agency, and Swedish Agency for Public Management as well as reports including wind coordinator statements and activities by local entities, such as the Energy Agency Southeast Sweden (Energikonto Sydost 2013), the Wind Power Centre of the Barents Region (Vindkraft Barents 2016), or the Municipality of Strömsund (Strömsunds Kommun 2011). The analysis was complemented by secondary literature focusing on the Swedish wind energy context (e.g., Söderholm and Petersson 2007, 2011; Granberg and Elander 2007; Bergek 2010; Wilkens et al. 2011; Larsson 2014; Wizelius 2014).

The study includes all four wind coordinators, since they at times act in unison when pushing for policy change. The analysis of entrepreneurial activities built on the classification presented by Roberts and King (1991), which was developed based on the public education sector. Similar to the implementation of wind power, the education field faces the challenge of national goals and local implementation with potential public resistance. The evaluation of these activities largely relies on a recent report published by the Swedish Statskontoret (Swedish Agency for Public Management 2016), which assesses wind power in general and the wind coordinator activities in particular.

When addressing the connection between entrepreneurial activities and policy change or policy outputs, it raises the question whether the output is static or dynamic (Capano and Howlett 2009). Since the wind coordinators have not been given concrete goals and are only one way for government to increase wind power levels among a change in legislation and other factors, it is difficult to make a direct link between the wind coordinators and wind power levels. This can be addressed by looking at the individual activities and breaking down the overall goal of increasing wind power into smaller goals, like optimizing wind turbine application procedures or conflict resolution over a specific wind turbine by a wind coordinator. It however remains a very dynamic set-up where stakeholders, interests and public opinions change or shift and the activities by the wind coordinators adjust over time.

## Findings

### Characteristics of wind power coordinators

Each of the four wind coordinators is in charge of several counties in one geographical area: Lars Thomsson is coordinating and facilitating projects in the areas of Stockholm, Uppsala; Västmanland, Västernorrland County; Örebro, Dalarna County; Gävleborg County; and Jämtland, which define the central region of Sweden. Stefan Lundmark is responsible for Västerbotten County and Norrbotten County. In Western Sweden, Lennart Värmby organizes the funding and project planning for wind turbines, which are supported by the EU and the Swedish government. His work also includes engaging the public in wind turbine planning. Agne Hansson facilitates networking in the southeastern part of Sweden. She is concerned with the planning and diminishing of bottlenecks for offshore wind power. This includes negotiations with government—jointly with other wind coordinators—on how to tax wind power (Swedish Energy Agency 2009; Energimyndigheten 2015).

All four wind coordinators have experiences with working for or with government and are connected to the region they are tasked to service. Since each coordinator only works in this capacity 25% of the time, they also hold other government (-related) positions. For example, Lars Thomsson lives in Gotland and between 2006 and 2010 was a counselor in charge of civil affairs. From 2007 to 2010, he was also the Building Committee Chairman in charge of building permits and implementation of wind power (Energimyndigheten 2015). Like Lars Thomsson, many of the wind coordinators have been politically active at national and/or local level in the energy field and are therefore familiar with the issues encountered in the municipalities they are responsible for. This gives them access to skills and competences within their personal network.

After establishing the wind coordinators in 2008, the Ministry of Environment and Energy started to invest 20 Million SEK per year into a network of wind power producers led by the Swedish Energy Agency. Based on this, the Swedish Energy Agency set up pilot projects to support the technical development of wind power in Sweden (IEA Wind 2013). The network is a collaboration of different government agencies, including municipalities and also energy and infrastructure organizations to ensure faster licensing and setting up of wind power facilities. The website Vindlov.se serves as the main communication tool by offering information about how to authorize and set-up wind turbines (Swedish Energy Agency 2015).

Taken together, the Ministry Swedish Agency for Public Management (Statskontoret) identifies three relevant groups of stakeholders in the wind energy field: The groups of actors involved in the licensing process, such as municipalities, county councils as well as judicial and consultative bodies. Another group consists of those that have expertise in the field of wind power, which include the Swedish Energy Agency, industry associations and universities. Finally, the third group consists of those that provide knowledge dissemination services to the wind energy stakeholders. Based on their mandate wind coordinators fall into this category, but as the following description of their activities will show, the coordinators have moved beyond dissemination services in the last couple of years.

### Wind coordinator activities

Wind coordinator activities are place-specific, but have similar characteristics. For example, the coordinators located in the South of Sweden deal more with offshore wind farms than those responsible for the North of the country (Swedish Energy Agency 2009). However, all of them take on the role of mediators among the diverse set of stakeholders involved in licensing and implementing wind power-related equipment. For example, wind coordinators participated in and have held seminars and meetings on wind power. These events were either used to connect a diverse set of stakeholders or specifically address issues with a particular wind turbine or wind farm. These types of meetings include the public as well as municipalities and energy providers (Invest in Norrbotten 2015). During this work, wind coordinators also connect with existing entities, such as the wind network, Vindlov.se, or Vindval, a cooperation between the Swedish Energy Agency and the Swedish Environmental Protection Agency (Swedish Energy Agency, 2009). They also have the task of supporting (municipal) planners in setting up and licensing wind turbines as well as reviewing the financial calculations made in connection to the particular turbines. Wind coordinators are described as having extensive mediation roles where they link various stakeholders, such as municipalities, local communities, and project managers (Statskontoret 2016). In the last 10 years, the individuals holding these positions have changed, as the coordinators responsible for the North of Sweden have been replaced. The current group of people has been active since 2011 (Regeringskansliet 2013; Energimyndigheten 2013).

#### Activities in line with mandate

Wind coordinators coordinate efforts among wind power companies and the Swedish municipalities they are assigned to. In fact, the recent evaluation report states that specifically those two wind coordinators working since 2006, Agne Hansson and Lennart Värmby, were able to form close ties with (local) authorities and thus helped to mediate among stakeholders (Statskontoret 2016). Wind coordinators further actively try to reduce obstacles by organizing wind energy events to bring different parties together. For example, the 2009 wind power and industry event in Kalmar, which was a meeting place for business contacts between the Swedish and international wind industry. It included 250 delegates from ten global wind turbine manufactures, 28 Swedish municipalities and organizations, and 130 Swedish companies. Another annual event is the national wind energy conference, which attracts around 200 participants from the Swedish wind energy sector (IEA Wind 2010).

To engage the public in wind power development projects, the wind coordinators further created contact points for coordination and dialogue as well as two indoor demonstrators at the science center in Karlshamn and a mobile center with an outdoor demonstrator (Energikonto Sydost 2013). Those facilities—actively supported and partly run by wind coordinators—help to deal with questions or requests communities might have as well as an overall information service for the region. These centers further offer guided study tours to wind parks and hold consultation meetings on possible wind turbine sites with the public. More specialized workshops, information meetings, and conferences can also be organized through these facilities (Energikonto Sydost 2013).

The Wind Power Centre of the Barents Region, established by the responsible wind coordinator Lundmark, for example, is based on the premise of establishing a hub when it comes to wind power in northern Sweden. The centre aims to be a meeting place for all stakeholders, to whom contractors, planners, private individuals, and municipalities can turn with their questions and comments (Vindkraft Barents 2016). Another initiative organized by the wind coordinators are the local wind power seminars held through the winter of 2010 to spring of 2011. In different locations across Sweden, those seminars were held in places interested in generating their own electricity through wind power. They also served as a platform for dialogue among municipality administration, politicians, and local stakeholders (Invest in Norbotten 2015).

#### Activities beyond mandate

Once the wind coordinators familiarized themselves with the local circumstances and established a relationship to the relevant stakeholders, they were able to take additional action. Wind coordinators have, for example, jointly pushed against a tax on wind cooperatives (Sveriges Riksdag 2010; Energimyndigheten 2015). Swedish municipalities have been involved in wind power since the early 1990s and have promoted wind power cooperatives. Municipalities invested into wind power cooperatives directly, for example Umea Energi, Skelleftea or Karlstad Energi (Wizelius 2014). They further have a model in which municipal real estate companies invest into turbines to produce power for certain regions which created a situation where no electricity tax needs to be paid, because the power is produced, transmitted, and finally used by people living in the neighborhood. Since then, energy companies, largely from other countries, have entered the market.

This increased the pace of wind power development, but also created regulatory tension over taxing of wind power facilities and led to a change in the tax regulation. Wind cooperatives now have to pay energy tax while companies do not (Wizelius 2014). The wind coordinators have pushed for an elimination of this tax (Energimyndigheten 2015). Together they lobbied at national level for its removal to stabilize the level of cooperative ownership. The reason behind this is the role that cooperatives play in the public support of wind turbines. A recent survey shows that “respondents preferred wind farms that are at least part-owned by either a cooperative or a local municipality, while private ownership was viewed much more negatively” (European Commission 2014, 1). The results further show that people are willing to make monetary trade-offs in order to ensure the involvement of the local population in the planning and implementing process, despite not being directly affected (Ek et al., 2013; European Commission 2014). The initiative by wind coordinators led to a legislative proposal [2009/10: Sk463] for abolishing the tax. It stated that since the 2008 tax, the conditions for cooperatives have worsened and that consequences have been severe for small operators in local communities who want to establish wind turbines. The proposal suggests flexible mechanisms for releasing financial resources to local wind energy productions while also offering a long-term investment and planning perspective. The legislative proposal was however dismissed in Swedish Parliament (Sveriges Riksdag 2010).

The coordinators further tried to improve the administrative processes connected to setting up wind energy. Stefan Lundmark, wind coordinator for Västerbotten County and Norrbotten County, criticized the lengthy environmental review authorities are engaged in. He aimed to cut the time for environmental assessments in half and pushed for changes while at the same time calling for a better balance between the economic and environmental aspects of wind turbines (Invest in Norrbotten 2015). Other wind coordinators have also been vocal about the environmental investigation conducted before the installation of wind turbines as well as the simplification of grid access for companies pursuing energy generation in the wind power field. Based on this, the threshold for the permission to set-up turbines was changed in 2009 from measurement by installed capacity to measurement by height and quantity (Wilkens, Johansson and Akesson 2011). In addition, there is now a legal obligation for all holders of grid concessions to connect anyone who wishes to be connected to the holder’s line on reasonable terms (Wilkens, Johansson and Akesson 2011). The hope is that this will make it easier for smaller companies pursuing wind power to afford a grid connection. Based on these changes, new projects were launched and are now being realized in different parts of Sweden (Strömsunds Kommun 2011).

#### Summary

Overall, the activities by wind coordinators have shifted since their establishment in 2006. According to the evaluation report (Statskontoret 2016), activities changed from knowledge dissemination to responding directly to concerns over the establishment of wind turbines and providing tailored information, bringing stakeholders together and mediating among them. This includes removing structural barriers to the environmental and permit processes by collaborating with authorities and organizations involved in setting up wind power facilities. As a final assessment, the recently published evaluation report states that coordinators have worked in different ways to promote the expansion of wind power in Sweden. The report acknowledges the coordinators’ role in handling problems with wind power implementation and helping to solve different types of obstacles linked to wind power expansion (Statskontoret 2016). It however remains difficult to specifically measure their impact on the increase in wind power, as there have also been regulatory changes that could have affected wind power developments.

The report concludes that the implementation of wind coordinators was a cost-effective way of facilitating wind power. A survey carried out by the evaluation authority Statskontoret (2016) shows that the different stakeholders involved in wind power implementation appreciate the work being undertaken by wind coordinators and speak to their continued mandate in the process. The report thus recommends a continued role for the four coordinators for the next three to four years in which the work description needs to be flexible and activities should not be extensively regulated (Statskontoret 2016).

## Discussion

The case shows that Swedish policy documents define a framework that determines the geographical space as well as the main activities of wind coordinators. These are elements described as “with mandate.” In the Swedish context, they largely include knowledge dissemination and bringing together relevant stakeholders at local level. Applying the theoretical framework by Roberts and King (1991), these are labelled as mobilization and execution activities. In the early phases, wind coordinators were able to collaborate with relevant stakeholders at national and local level. This also includes work with elected officials, coordination efforts, and the establishment of demonstration projects. This category also encompasses the local expertise that wind coordinators bring to their activities, which allows them to identify relevant problems and stakeholders.

However, as local wind coordinators took on those responsibilities, the activities broadened and shifted over time. Wind coordinators shaped their role in this context and moved from being sole knowledge disseminators and mediators towards innovating regulatory and bureaucratic guidelines for wind power implementation. Several factors contributed to this transition. First, the wind energy network, Vindlov.se, has gained momentum throughout the years, combining relevant information and actors in one place. This led wind coordinators to specialize more on regional issues in connection to wind turbines and pinpointing these issues also in the larger administrative procedures, such as legal or environmental assessments. Second, the transition was possible, because the role of wind coordinators was only loosely defined by the Ministry of Environment and Energy when establishing the position. In fact, this is assessed as one of the strengths by the recent evaluation report. So much so, that the report calls for limited regulation of the position in the future, giving the wind coordinators flexibility to adjust to local bottlenecks. Of the four theoretical categories defined by Roberts and Kind (1991), these activities are labeled as creative and intellectual, and administrative and evaluative activities, because wind coordinators were able to generate ideas, define problems, and facilitate program administration through lobbying for licensing changes for example. They seem to be largely contextually motivated, as wind coordinators learned about implementation and the different hurdles for the stakeholders in their network. These aspects of wind energy implementation are not mentioned in the policy documents and are thus labelled as “beyond mandate.”

Placing this analysis in the wider context of research on local policy entrepreneurs and their strategies, the paper contributes to the emerging literature on how context and conditions affect local entrepreneurs’ actions in policy implementation. Previous studies point towards the relevance of the network environment within which policy entrepreneurs operate, such as the characteristics of other network members, network complexity, and the relative power base of entrepreneurs (Fisher et al. 1983; De Bruijn and Ten Heuvelhof 2000; Stokman 1999). In addition, the policy framework plays a role, since it shapes the scope and interconnectedness of a project (Brouwer 2013). The Swedish case shows that a flexible framework allows emphasizing different activities over time, as networks and implementation issues develop. The interplay of local context and entrepreneurial activities highlights that as network relationships evolve; local entrepreneurs are able to identify and tackle issues arising in the implementation context. Finally, the entrepreneurial activities that exceed the mandate, in this case administrative/evaluative and creative/intellectual, are much more dependent on local knowledge of the implementation dynamics. Since they describe facilitating program administration and defining problems as well as selecting solutions, they require not only close collaboration with local stakeholders, but also a certain degree of understanding when it comes to the challenges linked to implementation. The mandated activities, mobilization, and execution, on the other hand, are more of relational nature in collaborating with key individuals and establishing a wider network around a project.

## Concluding remarks

The goal of this research is to contribute to the larger question of how context and conditions affect policy entrepreneur’s activities. By focusing on the local implementation of Swedish wind energy, the findings point towards policy entrepreneurs broadening their strategies as they identify issues through mediating and networking. The paper looks at the Swedish case of implementing wind coordinators to increase wind power capacity. The wind coordinators have the role of engaging with local stakeholders, such as municipalities, investors, and the public to put wind power sites in place. Wind coordinators have been active in knowledge dissemination on licensing of wind turbines, creating a network of stakeholders in wind energy implementation, and establishing demonstration projects to engage the public in this renewable energy source. Throughout these years, the coordinators were also able to further define their role and engage in entrepreneurial implementation activities that go beyond the position defined by Swedish government. They have had succeeded in easing local tensions over wind farms and putting forward suggestions for simplifying environmental assessment procedures. Wind coordinators further raised awareness for local bottlenecks in licensing of wind turbines. A recent evaluation report by the Swedish Agency for Public Management (2016) assesses the wind coordinators as a cost-effective and efficient way to support wind power implementation in Sweden and suggests additional funding for the next three to four years with a broad description of their role to allow flexibility in defining the role.

To identify the entrepreneurial activities carried out by the Swedish wind coordinators, the paper relies on the categories specified by Roberts and King (1991) and distinguishes between those activities defined by government and those arising out of the wind energy context. The four coordinators were able to act entrepreneurially by having a broad description of the role they were taking on in combination with their expertise on regulatory procedures, local issues, and stakeholders. Throughout the span of the time assessed (2006–2016), the coordinators re-defined their activities by moving beyond mobilization and execution activities to creative/intellectual and administrative/evaluative activities. These are largely anchored in place-specific initiatives that link to larger implementation issues, such as environmental assessment procedures. This poses the question for future research whether the freedom to shape the activities in a pre-defined public role facilitates entrepreneurial action and how closely this is linked to the (local) expertise of public employees for implementation success.

These findings are limited to the Swedish context in the area of wind energy implementation. This implies that the role of wind coordinators is rather unique in its form and definition. The study however draws out some of the circumstances in which local entrepreneurial action is shaped and poses the question for future research whether a temporal dimension in terms of personal experience of entrepreneurs with the specific project and the structural definition of the position affect the innovation strategies being chosen. In short, whether entrepreneurial action shifts from networking and mediating towards finding innovative policy solutions as local knowledge about implementation is accumulated.

## Footnotes

1 The directive applies to two or more turbines that are taller than 150 m or seven or more plants that are taller than 120 m.
